# Improve the diagnosis of idiopathic normal pressure hydrocephalus by combining abnormal cortical thickness and ventricular morphometry

**DOI:** 10.3389/fnagi.2024.1338755

**Published:** 2024-02-29

**Authors:** Yifeng Yang, Meijing Yan, Xiao Liu, Shihong Li, Guangwu Lin

**Affiliations:** Department of Radiology, Huadong Hospital Affiliated to Fudan University, Shanghai, China

**Keywords:** idiopathic normal pressure hydrocephalus (iNPH), brain morphometry, machine learning, multi-feature fusion, diagnosis

## Abstract

**Background:**

The primary imaging markers for idiopathic Normal Pressure Hydrocephalus (iNPH) emphasize morphological measurements within the ventricular system, with no attention given to alterations in brain parenchyma. This study aimed to investigate the potential effectiveness of combining ventricular morphometry and cortical structural measurements as diagnostic biomarkers for iNPH.

**Methods:**

A total of 57 iNPH patients and 55 age-matched healthy controls (HC) were recruited in this study. Firstly, manual measurements of ventricular morphology, including Evans Index (EI), z-Evans Index (z-EI), Cella Media Width (CMW), Callosal Angle (CA), and Callosal Height (CH), were conducted based on MRI scans. Cortical thickness measurements were obtained, and statistical analyses were performed using surface-based morphometric analysis. Secondly, three distinct models were developed using machine learning algorithms, each based on a different input feature: a ventricular morphology model (LVM), a cortical thickness model (CT), and a fusion model (All) incorporating both features. Model performances were assessed using 10-fold cross validation and tested on an independent dataset. Model interpretation utilized Shapley Additive Interpretation (SHAP), providing a visualization of the contribution of each variable in the predictive model. Finally, Spearman correlation coefficients were calculated to evaluate the relationship between imaging biomarkers and clinical symptoms.

**Results:**

iNPH patients exhibited notable differences in cortical thickness compared to HC. This included reduced thickness in the frontal, temporal, and cingulate cortices, along with increased thickness in the supracentral gyrus. The diagnostic performance of the fusion model (All) for iNPH surpassed that of the single-feature models, achieving an average accuracy of 90.43%, sensitivity of 90.00%, specificity of 90.91%, and Matthews correlation coefficient (MCC) of 81.03%. This improvement in accuracy (6.09%), sensitivity (11.67%), and MCC (11.25%) compared to the LVM strategy was significant. Shap analysis revealed the crucial role of cortical thickness in the right isthmus cingulate cortex, emerging as the most influential factor in distinguishing iNPH from HC. Additionally, significant correlations were observed between the typical triad symptoms of iNPH patients and cortical structural alterations.

**Conclusion:**

This study emphasizes the significant role of cortical structure changes in the diagnosis of iNPH, providing a novel insights for assisting clinicians in improving the identification and detection of iNPH.

## 1 Introduction

Idiopathic normal-pressure hydrocephalus (iNPH) is a hydrocephalus syndrome characterized by enlarged ventricles and normal cerebrospinal fluid pressure levels. Common symptoms include cognitive impairment, gait disturbance, and urinary incontinence. Despite its unknown etiology (Relkin et al., 2005; Nakajima et al., 2021), iNPH stands out from other neurodegenerative diseases due to the potential reversibility of its symptoms. In clinical practice, the cerebrospinal fluid tap test (CSF-TT) or external lumbar drainage (ELD) surgery serves as valuable tools for the detection of iNPH. Targeted interventions like shunt surgery and ventriculocisternostomy can significantly improve patient conditions (Popal et al., 2021). In particular, iNPH-associated dementia is currently recognized as the first treatable form of dementia (Ishikawa et al., 2012). However, the clinical diagnosis of this treatable dementia is a subject of widespread debate. Identifying at-risk individuals is challenging, leading to only a minority of iNPH patients receiving timely and appropriate treatment (Abu Hamdeh et al., 2018; Kockum et al., 2020; Hua et al., 2021). Consequently, there is an urgent need for robust and reliable biomarkers that can facilitate the screening and identification of individuals at high risk for developing iNPH.

Due to the lack of reliable non-invasive diagnostic techniques, the definitive diagnosis of iNPH often hinges on the outcomes of cerebrospinal fluid shunting procedures. In recent years, radiological features have gained increasing importance in diagnosing and assessing iNPH (Ryska et al., 2020). Brain imaging markers with potential in distinguishing iNPH from other conditions and predicting the efficacy of cerebrospinal fluid shunting include the Evans Index (EI), Disproportionate Expansion of Subarachnoid Space Hydrops (DESH), Corpus Callosum Angle (CA), and various measurements of ventricular size, shape, and volume (Ryska et al., 2020; Park et al., 2021). However, these markers exhibit variations in diagnostic sensitivity and specificity, leading to inconsistent implementation and interpretation in clinical settings. While several studies support DESH as a predictor of shunt success, approximately 6% of iNPH cases without DESH show symptomatic improvement after cerebrospinal fluid tapping, raising doubts about the consistency of this marker as a predictive tool (Narita et al., 2016; Agerskov et al., 2019). Concerning EI, a value of 0.3 or above is required for iNPH diagnostic criteria, yet Brix et al. demonstrated that this threshold may also be reached in healthy older adults (Brix et al., 2017). On the other hand, CA is considered the most accurate single imaging marker for iNPH detection. However, Fällmar et al. found that a combined scale, including EI, CA, enlarged sulcus, and Sylvian fissure width, showed improved diagnostic accuracy (Fällmar et al., 2021). These studies underscore that two-dimensional ventricular morphometry for quantifying ventricular enlargement is nonspecific and insufficient as independent diagnostic or prognostic indicators for iNPH. Moreover, it fails to explain the typical triad symptoms of iNPH patients (Espay et al., 2017; Mantovani et al., 2020; Pyrgelis et al., 2023).

The cortex, the outer layer of the cerebral gray matter responsible for controlling higher cognitive functions, is a crucial component of the brain. Surface-based morphological (SBM) measurement of cortical thickness has emerged as a precise and powerful tool for detecting local atrophy. It has been widely applied in studying the structural characteristics of the brain in various neurodegenerative diseases (Hutton et al., 2008; Kang et al., 2018). Previous researches in animal models and children with hydrocephalus has reported an association between increasing ventriculomegaly and more marked thinning of the cerebral cortex (Olopade et al., 2012; Pyrgelis et al., 2023). A study revealed that total cortical thickness was thinner in the iNPH group compared with the control group (Moore et al., 2012). Kang et al. (2018) observed a correlation between increased ventricular size and decreased cortical thickness across different brain regions, including the prefrontal, temporal, parietal, and occipital cortices, as well as subcortical structures like the hippocampus and thalamus. These findings suggest that cortical thinning is also a potential feature of iNPH and may indicate underlying neural damage or degeneration.

However, the precise causes driving cortical thickness changes in iNPH are yet to be elucidated. Some studies attribute the alterations to abnormal cerebrovascular blood flow and hypoxia (Kang et al., 2020, 2023), while others propose that neuroinflammation or neurodegeneration may be involved (Gao et al., 2013; Ferraro et al., 2018; Montal et al., 2018). Additionally, the relationship between cortical thickness changes and iNPH symptoms has gained attention. For instance, decreased thickness in the anterior cingulate gyrus has been associated with executive dysfunction, while decreased thickness in the precuneus has been linked to gait disturbances (Kang et al., 2020). Moreover, patterns of cortical thinning have shown promise as potential predictor of response to shunt surgery in patients with ventriculomegaly (Kang et al., 2013). Although cortical thickness changes present a promising avenue for exploring iNPH pathogenesis and optimizing treatment approaches, reliable biomarkers for the diagnosis and prognosis of iNPH-induced cortical thinning remain elusive.

The aim of this study was to assess the value of integrating altered brain parenchyma and ventricular morphology features to improve the diagnosis of iNPH. We firstly employed surface-based morphometric analysis to mine features of cortical structural alterations associated with iNPH. Subsequently, three distinct models for iNPH identification were developed, utilizing machine learning algorithms based on three different input features: a ventricular morphology-only model (LVM), a cortical thickness-only model (CT), and a fusion model combining both (All). The significance of cortical structural alterations in iNPH diagnosis was systematically investigated by evaluating the performance of the models for the identification of iNPH from HC. This evaluation also included a comparison of the contribution of each feature and an analysis of the correlation with clinical manifestations. This research seeks to find novel imaging markers that would improve the precision and efficiency of diagnosing iNPH, thereby reducing dependence on invasive and costly diagnostic methods.

## 2 Materials and methods

### 2.1 Participants and clinical symptoms assessments

This study was approved by the Medical Ethics Committee of Huadong Hospital Affiliated to Fudan University (approval number: 2017 K027), which authorized a retrospective analysis of eligible patients between August 2016 and August 2020, and the informed consent of the subjects was exempted. Inclusion criteria for iNPH were established according to the guidelines of Experts consensus on diagnosis and treatment of normal pressure hydrocephalus in China (2016). The inclusion criteria were as follows: (1) age of 60 years or older; (2) presentation of at least 1 of the following: gait disturbance, dementia, urinary incontinence; (3) ventricular enlargement (Evans Index >0.3) with narrowing of the subarachnoid space over the high-convexity (DESH); (4) CSF pressure < 200 mmH2O and normal CSF content; (5) improvement of symptoms after CSF-TT and shunt surgery. Exclusion criteria included: (1) cerebral infarction and dementia caused by clear causes and hospitalization for severe mental illness; (2) preceding diseases possibly causing ventricular dilation (including subarachnoid hemorrhage, meningitis, head injury, congenital/developmental hydrocephalus, and aqueductal stenosis); (3) secondary normal pressure hydrocephalus due to stroke, trauma, hemorrhage, or meningitis. Inclusion criteria for healthy controls (HC) included: (1) matched age to iNPH group; (2) no abnormalities were observed with conventional head MRI; (3) no any active neurological, systemic, or psychiatric disorders.

For all iNPH patients, clinical symptoms were examined by two trained neurologists prior to performing CSF-TT, including routine neurologic examination and the cognitive function was tested with the Mini-Mental State Examination (MMSE). The overall severity of iNPH was adequately evaluated by the used iNPH grading scale (iNPHGS) as a subjective evaluation scale (Kubo et al., 2008). The iNPHGS adequately evaluated the overall severity of iNPH based on three primary clinical signs: cognitive impairment, gait difficulties, and urinary dysfunction. Each symptom was rated on a scale of 0 (none) to 4 (severe), producing an overall score ranging from 0 to 12 where higher scores denote increasing severity. Separate scales, cognitive-iNPHGS (r-iNPHGS), gait-iNPHGS (g-iNPHGS), and urinary control-iNPHGS (u-iNPHGS), were used to assess cognitive function, walking ability, and urinary dysfunction, respectively.

### 2.2 MRI acquisition

All subjects underwent MRI scans using the Prisma 3.0 T scanner from Siemens in Germany. The scanning process involved a 32-channel head coil and a gradient field strength of 50 mT/T. The scanning sequence primarily included sagittal T1-weighted magnetization-preparatory rapid acquisition gradient echo (T1-MPRAGE) and T2 fluid-attenuated inversion recovery imaging (T2-FLAIR). The axial positioning line was parallel to the AC-PC line, and the sagittal positioning line was parallel to the mid-sagittal plane, with a scan range covering the foramen magnum to the vertex. The parameters for T1-MPRAGE were TR = 1800 ms, TE = 2.37 ms, FOV = 250 mm × 250 mm, number of excitations = 1, slice thickness = 0.85 mm, gap = 50%, scanning time = 219 s, total slices = 208. Coronal images perpendicular to the AC-PC level were obtained by multiplanar reconstruction from sagittal T1-MPRAGE. Conventional MRI protocols were obtained to identify any other brain abnormalities.

### 2.3 Measurements of lateral ventricle morphology

The study focused on measuring eight imaging metrics commonly utilized for lateral ventricular morphometry (LVM) in diagnosing iNPH, as outlined in prior research (Ryska et al., 2020). These measurements were conducted in three planes: transverse, sagittal, and coronal, which intersecting the anterior–posterior commissure and perpendicular to the anterior–posterior commissure line, as illustrated in Supplementary Figure S1. The Evans Index (EI), a ratio of the maximal frontal horn diameter (FHD) to the inner skull diameter (ISD), was measured in the transverse plane. Callosal height (CH), callosal-commissural distance (CCD), and maximum supratentorial intracranial diameter (MSID) were measured in the median sagittal plane. Frontal horn vertical diameter (FHVD), representing the anterior association to the lateral ventricles, was measured in the coronal plane perpendicular to the anterior–posterior joint connection and passing through the anterior commissure. zEI, the ratio of FHVD to MSID, reflects the relationship between the lateral ventricle and the effect of the discharge test in iNPH patients in the Z-axis direction. Callosal angle (CA), the inner wall angle of the lateral ventricle, was measured in the coronal plane perpendicular to the anterior and passing through the posterior joint connection. In the same plane, callosal media width (CMW) was quantified as the vertical distance from the top of the lateral ventricle to the bottom. Additionally, callosal ventricular distance (CVD) denoted the distance between the line that connects the tops of the ventricles and the bottommost point of the corpus callosum. For symmetric structures such as lateral ventricles, mean measurements on both sides were calculated as morphological features. Two experienced radiologists independently conducted the blind visual assessment. Inter-rater agreement was assessed using the Cohens kappa (κ = 0.84), ensuring the accuracy of the clinical diagnosis.

### 2.4 Cortical thickness analysis

We employed the CAT12 toolset (version r1727), an open-source expansion built upon SPM12^1^ compatible with MATLAB 2018b,^2^ to preprocess 3D MRI data and measure cortical thickness. Initially, the images were aligned to the MNI-152 standard anatomical space of the Montreal Institute of Neurology. They were then segmented into gray matter (GM), white matter (WM), and cerebrospinal fluid. The intensity values in the segmented gray matter images were subsequently corrected. Then, automated brain surface reconstruction and cortical thickness estimation were performed by projection based thickness (PBT) method (Dahnke et al., 2013). The procedure included registering the cortical surface, increasing the subdivision of folded areas, and automatically rectifying topological abnormalities. All images underwent a homogeneity check for image quality assessment and to ensure consistent quality. In order to improve the signal-to-noise ratio of the image, a spatial smoothing kernel measuring 15 mm was utilized for smoothing purposes.

The two-sample *t-*test was conducted to compare the differences in cortical thicknesses across entire cortex vertices between the two groups, while controlling for age and gender as covariates. Cluster-level family-wise error (FWE) correction was applied with a significance threshold of *p* < 0.05. The Desikan-Killiany atlas template was employed to label the cortical areas where significant clusters were located (Klein and Tourville, 2012). Subsequently, the mean cortical thickness of all vertices within each cluster was computed as the cortical thickness feature for the specific brain region.

### 2.5 Model construction

We assessed the independent discriminatory capability of each image feature in distinguishing iNPH from HC using a simple logistic regression algorithm. Then, classifiers were constructed based on three distinct combinations of imaging traits: LVM (combined ventricular morphometry), CT (combined cortical thickness measures), and All (combined LVM and CT). These were designed to investigate whether feature combinations could improve the ability to differentiate between iNPH and HC.

The dataset was stratified and divided into two parts, with 80% used as the training set and 20% as the independent test set. This random division aimed to facilitate the process of feature selection and model training, which relied on the training set. To gauge the discriminatory power of individual imaging features against iNPH cases, receiver operating characteristic (ROC) curves were plotted, and corresponding area under the curve (AUC) scores were calculated from the independent test set, allowing for a comparative analysis.

For the assessment of three different feature combination methods, the Least absolute shrinkage and selection operator with cross-validation (LassoCV) method was initially employed for feature selection. This method can handle potential correlations between features while reducing feature dimensions to minimize collinearity (Ranstam and Cook, 2018; Jin et al., 2023). Next, eight state-of-the-art machine learning algorithms were utilized to build models based on three different feature combinations, respectively. These models included Decision Tree (DT), Logistic Regression (LR), Gaussian Naive Bayes (GaussainNB), Random Forest (RF), Support Vector Machine (SVC), Multilayer Perceptron (MLP), Adaptive Boosting (AdaBoost), and XGBoost (XGB). To identify an appropriate algorithm to serve as the foundation for subsequent comparisons, default parameters were set for all algorithms. An internal stratified 10-fold cross-validation approach on the training data was implemented to assess the capabilities of these diverse machine learning algorithms. By computing the average values of four key performance indicators–accuracy, sensitivity, specificity, and Matthews correlation coefficient (MCC)–to identify the best classifier algorithm among them. Among them, MCC is a comprehensive measure used to assess the quality of binary classifications. It takes into consideration true positives, true negatives, false positives, and false negatives, making it a balanced evaluation metric (Chicco and Jurman, 2020). MCC ranges from −1 to +1, where +1 represents perfect prediction, −1 indicates total disagreement, and 0 implies no better than chance prediction. The formula was as follows:


$$\begin{array}{c} MCC=\left(\left({TP}^{*}TN\right)-\left({FP}^{*}FN\right)\right)\\ /\mathrm{sqrt}\left({\left(TP+FP\right)}^{*}{\left(TP+FN\right)}^{*}{\left(TN+FP\right)}^{*}\left(TN+FN\right)\right) \end{array}$$

Where TP, TN, FP, FN represent true positive, true negative, false positive, and false negative, respectively.

Ultimately, leveraging the identified optimal classifier algorithm, we conducted model optimization using 10-fold cross-validation and grid search on the training set. Subsequently, we separately evaluated and compared the average prediction performance of three distinct feature combination methods on the independent test set. In addition, the decision curve analysis (DCA), ROC, precision/recall (P-R) curve, and calibration curve were plotting to assess the clinical utility and the reliability of the predictive probabilities for models.

### 2.6 Statistical analysis

Statistical analyses were carried out using IBM SPSS version 26.0 (SPSS Inc.). Differences in sex distribution between groups were assessed using the chi-square test (*X*^2^ test). Age and clinical assessment data were compared between patients with iNPH and HC using the non-parametric Mann–Whitney *U* test after testing for normality assumptions. Ventricular morphometric markers were contrasted using two-tailed t-tests. Model performance was compared using ANOVA tests, followed by *post hoc* comparisons (Bonferroni-corrected *p* < 0.05). In the clinically diagnosed iNPH group, Spearman correlation coefficients were computed to explore relationships between clinical symptom scores and various imaging markers. Statistical significance was defined as *p* < 0.05 (two-tailed).

The Shapley Additive Explanations (SHAP) technique was ultimately employed to identify the risk factors that are most closely associated with iNPH, using the model that demonstrated the highest level of performance (Lundberg and Lee, 2017). The visual display of the SHAP value demonstrated the significance and impact of each feature on iNPH.

The experiments in our study were performed with the following configurations: 32GB with RAM, Core i7-8700 CPU, NVIDIA GeForce GTX® 2080 Ti GPU and PyCharm 2018.3.5 × 64 as the programming platform for Python 3.8. Further, other open-source libraries include scikit-learn, SHAP, etc.

## 3 Results

### 3.1 Patient characteristics

Table 1 presents an overview of baseline demographic statistics for both two cohorts, including 57 iNPH cases and 55 healthy controls. There were no significant differences observed in terms of age or gender between the two groups. Additionally, iNPH patients had an average total score of 7.43 on the iNPHGS scale, along with an average MMSE score of 16.6. There were generally significant differences in lateral ventricular morphology measurements between the two groups (*p* < 0.001).

**Table 1:** Demographic information for both the iNPH and HC participants.

	iNPH (*n* = 57)	HC (*n* = 55)	*p*-value
Sex (male/female)	40/17	30/25	0.088
Age (years)	74.00 ± 7.81	71.94 ± 6.67	0.134
Clinical Symptom			
r-iNPHGS	2.59 ± 0.88	/	/
g-iNPHGS	2.62 ± 0.64	/	/
u-iNPHGS	2.22 ± 1.17	/	/
iNPHGS	7.43 ± 2.23	/	/
MMSE	16.60 ± 8.49	/	/
Lateral ventricular morphometry			
EI	0.32 ± 0.04	0.26 ± 0.03	<0.001***
CA	88.24 ± 22.56	118.03 ± 11.57	<0.001***
FHVD	3.51 ± 0.48	2.69 ± 0.34	<0.001***
CMW	2.40 ± 0.55	1.26 ± 0.43	<0.001***
CVD	1.19 ± 0.37	0.89 ± 0.21	<0.001***
CCD	3.04 ± 0.46	2.32 ± 0.39	<0.001***
zEI	0.37 ± 0.05	0.28 ± 0.03	<0.001***
CH	2.55 ± 0.37	1.99 ± 0.33	<0.001***

Values denote the mean ± SD. iNPH, idiopathic normal-pressure hydrocephalus; HC, healthy control; iNPHGS, idiopathic normal-pressure hydrocephalus grading scale; r-iNPHGS, Cognitive-iNPHGS; g-iNPHGS, Gait-iNPHGS; u-iNPHGS, Urinary Control-iNPHGS; MMSE, Mini-Mental Status Examination; EI, Evans index; CA, callosal angle; FHVD, frontal horn vertical diameter; CMW, callosal media width; CVD, callosal ventricular distance; CCD, callosal-commissural distance; zEI: z-Evans index; CH, callosal height. _***_p < 0.001.

### 3.2 Group differences in cortical thickness

**Figure 1:**
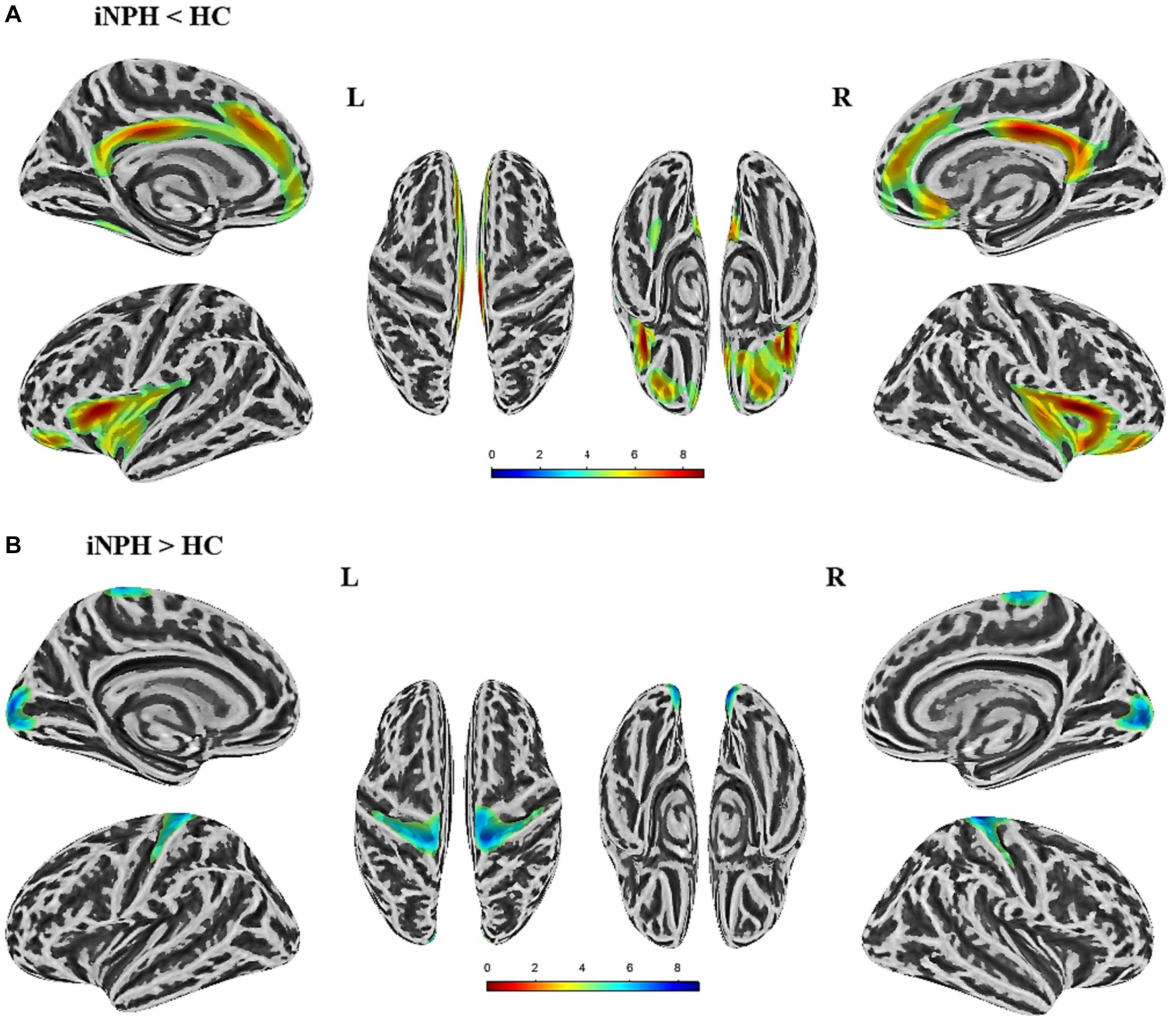
Significant differences in cortical thickness between iNPH patients and healthy controls (HC) (*p* < 0.05,FWE corrected). **(A)** The brain regions where cortical thickness is significantly reduced in iNPH patients compared to HC. **(B)** The brain regions where cortical thickness is increased in iNPH patients compared to the HC. L, left; R, right; FWE, family-wise error.

The surface-based morphological analysis uncovered noteworthy dissimilarities in the cortical thickness of nine cortex clusters between the two populations, corrected with multiple comparisons (cluster-level *p* value <0.05, vertex-level *p* value <0.001) (Figure 1). As shown in Table 2, individuals with iNPH exhibited thinner cortices in five clusters compared to the HC. Specifically, four of these clusters centered in the bilateral insula, superior frontal gyrus, and isthmus cingulate cortex, along with one cluster located in the left fusiform gyrus. On the other hand, there was increased cortical thickness in the bilateral postcentral gyrus and pericalcarine sulcus among the iNPH cohort compared to controls.

**Table 2:** Clusters of significant differences in cortical thickness between iNPH patients and HCs.

Comparison	Hemisphere	Cluster-Size (vertexes)	Peak-level *t-*value (*p-*value)	Overlap of atlas region	Brain region
iNPH < HC	lh	6,492	8.6 (<0.001)	55%	Insula
				13%	Pars triangularis
				11%	Pars opercularis
		5,644	7.5 (<0.001)	30%	Superior frontal
				20%	Posterior cingulate
				20%	Isthmus cingulate
				12%	Caudal anterior cingulate
				11%	Medial orbitofrontal
		500	5.2 (<0.001)	100%	Fusiform
	rh	10,439	8.8 (<0.001)	38%	Insula
				26%	Lateral orbitofrontal
		3,585	8.0 (<0.001)	53%	Isthmus cingulate
				35%	Posterior cingulate
				12%	Precuneus
iNPH > HC	lh	4,585	6.5 (<0.001)	61%	Postcentral
				20%	Paracentral
				20%	Precentral
		1,593	7.3 (<0.001)	31%	Pericalcarine
				29%	Cuneus
				22%	Lateral occipital
				19%	Lingual
	rh	4,238	7.1 (<0.001)	51%	Postcentral
				27%	Precentral
				21%	Paracentral
		1,780	7.0 (<0.001)	55%	Pericalcarine
				18%	Lingual
				17%	Cuneus
				10%	Lateral occipital

Corrected for multiple comparisons using the peak-level FWE at a threshold of *p* < 0.001. Key brain regions accounting for more than 10% of voxels were listed. lh, left hemisphere; rh, right hemisphere.

### 3.3 Correlations of ventricular expansion and cortical changes

**Figure 2:**
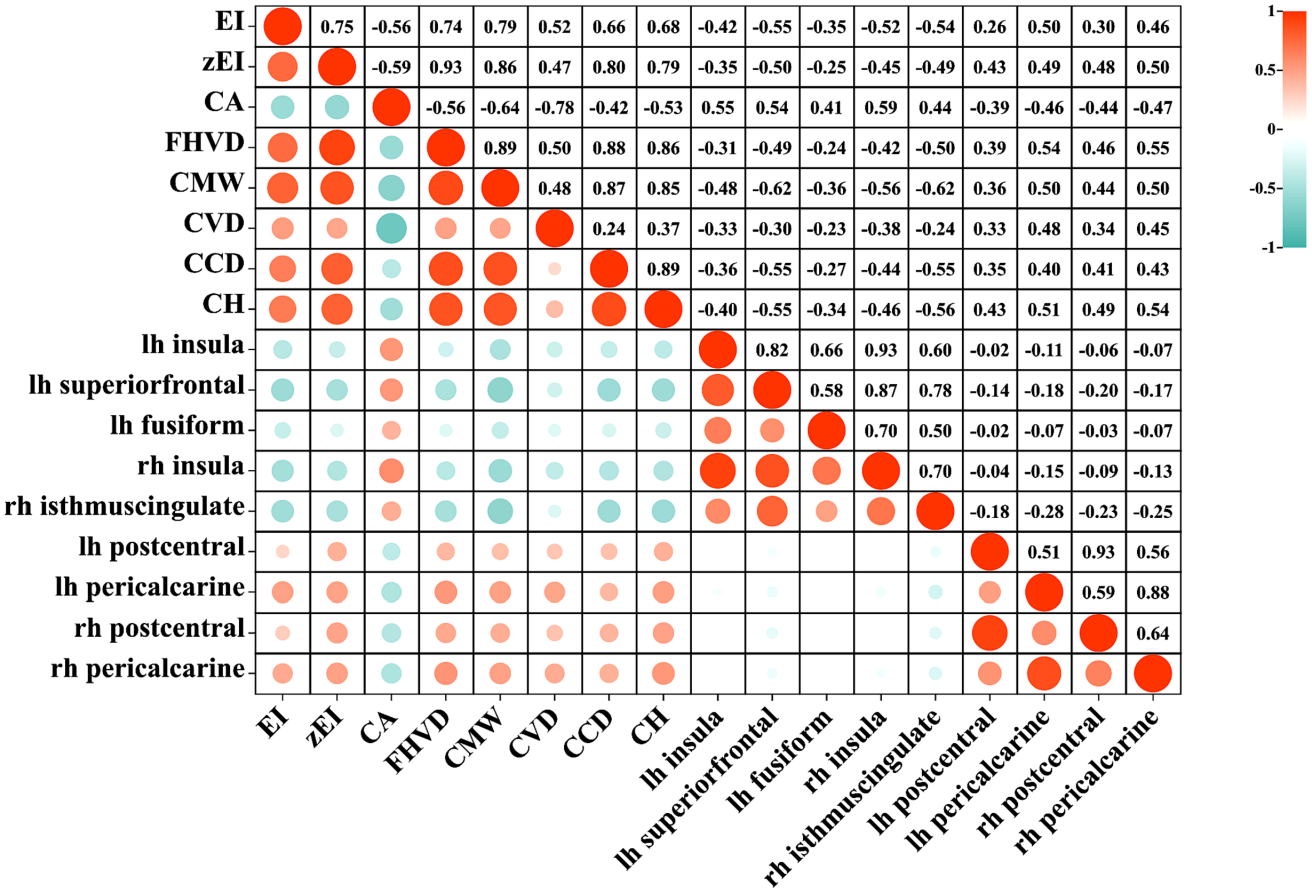
The heatmap of correlation between lateral ventricular expansion and cortical thickness. EI, evans index; z-EI, z-evans index; CA, callosal angle; FHVD, frontal horn vertical diameter; CMW, cella media width; CVD, callosal ventricular distance; CCD, callosal-commissural distance; CH, callosal height; lh, left hemisphere; rh, right hemisphere.

Figure 2 showed the correlation between cortical thickness in significant clusters and ventricular morphological measurements. A strong correlation was found between ventricular dilation and cortical changes. For example, there was a significant negative correlation between EI and the thinning of the left superior frontal cortex (*r* = −0.54, *p* < 0.001), as well as with zEI (*r* = −0.50, *p* < 0.001), CMW (*r* = −0.62, *p* < 0.001), and CCD (*r* = −0.55, *p* < 0.001). CH had a significant negative correlation with the thinning of the right isthmus cingulate (*r* = −0.56, *p* < 0.001). On the other hand, CA angle had a significant positive correlation with the thinning of the right insula region (*r* = 0.59, *p* < 0.001). FHVD had a significant positive correlation with the thickening of the right pericalcarine (*r* = 0.55, *p* < 0.001).

### 3.4 Classification performance

**Figure 3:**
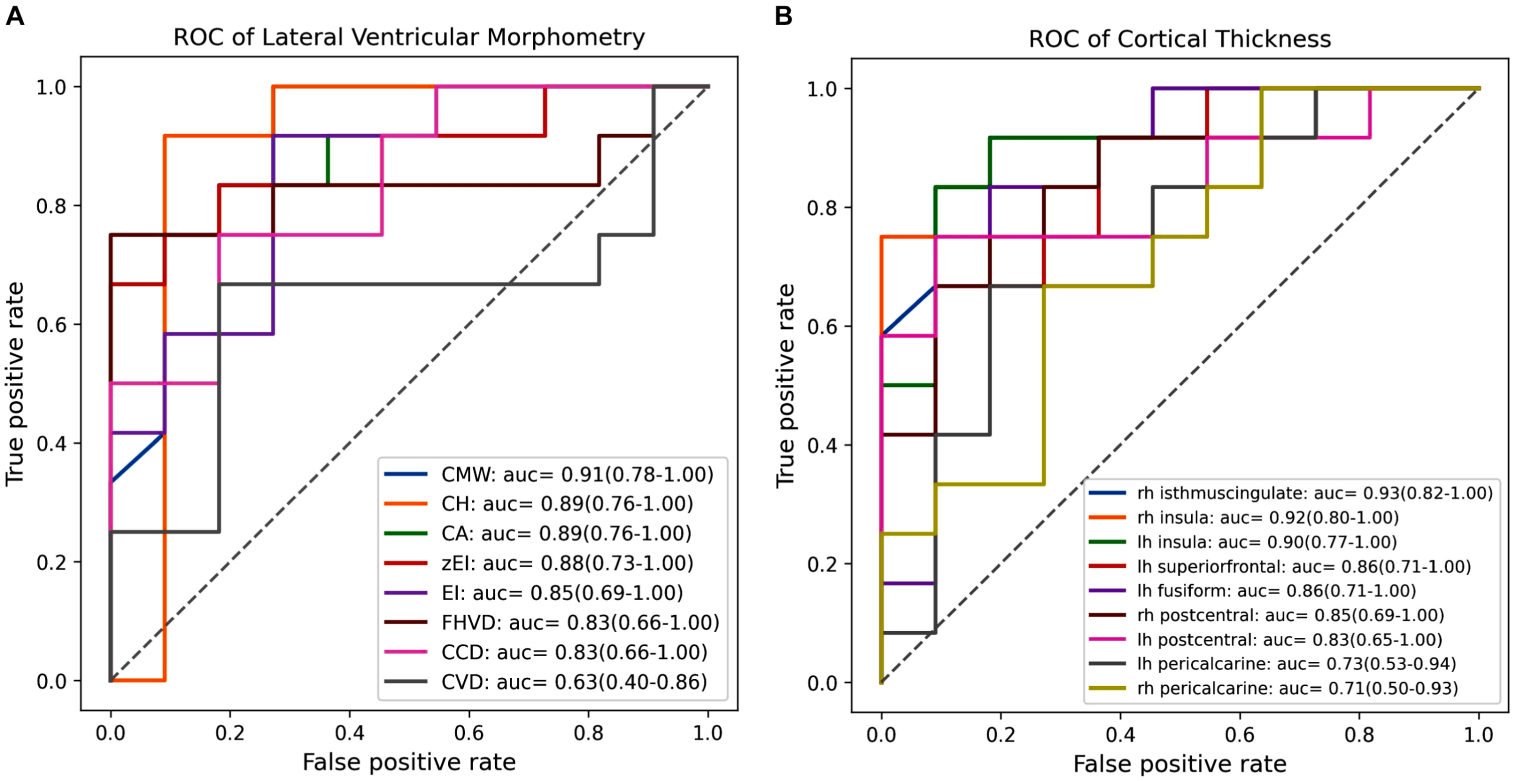
The receiver operating characteristic (ROC) curves and area under the curve (AUC) values of each feature in individually diagnosing iNPH. **(A)** The ROC curve and AUC value for each lateral ventricular morphological feature. **(B)** The ROC curve and AUC value for each cortical thickness within significant clusters. EI, evans index; z-EI, z-evans index, CA, callosal angle; FHVD, frontal horn vertical diameter; CMW, cella media width; CVD, callosal ventricular distance; CCD, callosal-commissural distance; CH, callosal height; lh, left hemisphere; rh, right hemisphere.

Figure 3 displayed the efficacy of each imaging feature for diagnosing iNPH individually, determined through evaluation on independent test set with LR classifier. Among lateral ventricular morphometric features, CMW emerged as the most effective predictor of iNPH status, achieving AUC of 0.91with a 95% confidence interval ranging from 0.78 to 1.00. Following close behind were CH and CA, all of which achieved an AUC score of 0.89 and a corresponding 95% CI ranging from 0.76 to 1.00. In contrast, for cortical thickness metrics, the isthmus cingulate cortex in the right hemisphere proved to be the strongest indicator of iNPH, achieving an AUC score of 0.93 and a 95% CI ranging from 0.82 to 1.00. Not far behind was the insula cortex in the right hemisphere, which also obtained a higher AUC score of 0.92 but with a 95% CI ranging of 0.80 to 1.00. Collectively, these results highlighted the potential value of lateral ventricular morphometry and specific cortical-related features for accurate diagnosis of iNPH.

To fairly evaluate the diagnostic capabilities of the three various fused feature sets (LVM, CT, and All), we applied LassoCV-based feature selection methods to identify optimal combinations of variables within each fused feature set, as illustrated in Supplementary Figures S2, S3. A total of six essential morphometry parameters were ultimately chosen among lateral ventricular morphometry measures: CMW, CA, zEI, CVD, EI, and CH. Similarly, seven pivotal traits were identified among cortical thickness indices, including rh isthmus cingulate, lh pericalcarine, rh insula, lh superior frontal, rh pericalcarine, and lh insula. Finally, in the comprehensive feature set (All), 11 features were deemed critical: CMW, FHVD, lh insula, rh isthmus cingulate, CH, CA, lh superior frontal, lh postcentral gyrus, zEI, and rh pericalcarine gyrus.

**Figure 4:**
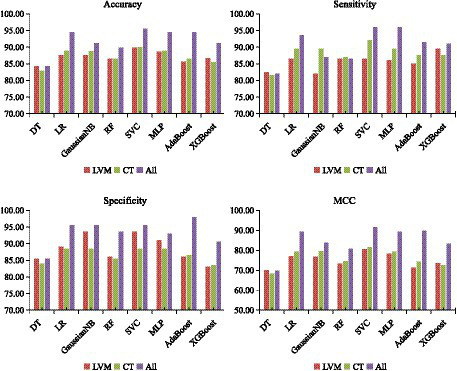
Classification performance of eight classifiers across three features combinations and 10-fold cross-validation on training set. DT, decision tree; LR, logistic regression; RF, random forest; SVC, support vector machine; MLP, multilayer perceptron; AdaBoost, adaptive boosting; XGB, eXtreme gradient boosting. LVM, lateral ventricular morphometry; CT, cortical thickness measures; All, combined LVM & CT; MCC, Matthews correlation coefficient.

Following the optimization of feature subsets as outlined above, the average classification performance of eight classifiers using three features combination methods were demonstrated in Figure 4. This assessment was carried out through 10-fold cross-validation within the training set. Overall, the classification performance of the SVC algorithm outperformed that of other classifier algorithms across all three different feature inputs. Particularly noteworthy was the comprehensive evaluation metric MCC value, consistently more than 80%. As a result, the SVC algorithm was identified as the effective classification algorithm in this study.

Using SVC classification algorithm, we defined the hyperparameter optimization range as follows: Kernel: [‘linear’, ‘poly’, ‘rbf’]; C: [0.0001,0.001,0.01,0.1,1]; gamma: [‘scale,’ ‘auto,’ 0.1,1]. Following grid parameter optimization, the optimal parameter combination for the SVC algorithm was determined with the kernel set to ‘linear’, C equaled to 0.001, and gamma set to ‘scale.’ As outlined in Table 3, during the internal evaluation on the training set, the model achieved the highest classification accuracy (95.56% ± 0.54%), sensitivity (96.00% ± 0.64%), specificity (95.50% ± 0.82%), and MCC values (91.50% ± 2.10%). Moreover, on the independent test set, the model employing the All features input strategy demonstrated an average accuracy of 90.43%, sensitivity of 90.00%, specificity of 90.91%, and an MCC value of 81.03%. Interestingly, while the model utilizing only the CT feature combination strategy excelled in terms of sensitivity, its specificity was relatively subpar than that of LVM based model. However, a balance between sensitivity and specificity metrics was achieved by the model based on all feature inputs.

**Table 3:** Average accuracy, sensitivity, specificity, and MCC of each model on the internal 10-fold cross-validation of the training set and the independent test set.

	LVM		CT		All	
	Training set	Testing set	Training set	Testing set	Training set	Testing set
Accuracy (%)	89.86 ± 1.10	84.34 ± 0.27	90.00 ± 1.10	88.89 ± 1.23	95.56 ± 0.54	90.43 ± 0.11
Sensitivity (%)	86.50 ± 2.50	78.33 ± 1.00	92.00 ± 1.76	89.50 ± 1.92	96.00 ± 0.64	90.00 ± 0.39
Specificity (%)	93.50 ± 1.00	90.91 ± 0.00	88.50 ± 2.60	88.50 ± 2.60	95.50 ± 0.82	90.91 ± 0.00
MCC (%)	80.41 ± 4.57	69.78 ± 0.88	81.48 ± 4.01	78.98 ± 1.21	91.50 ± 2.10	81.03 ± 0.13

Values were presented as mean ± SD. LVM, lateral ventricular morphometry; CT, cortical thickness measures; All, combined LVM and CT; MCC, Matthews correlation coefficient.

**Figure 5:**
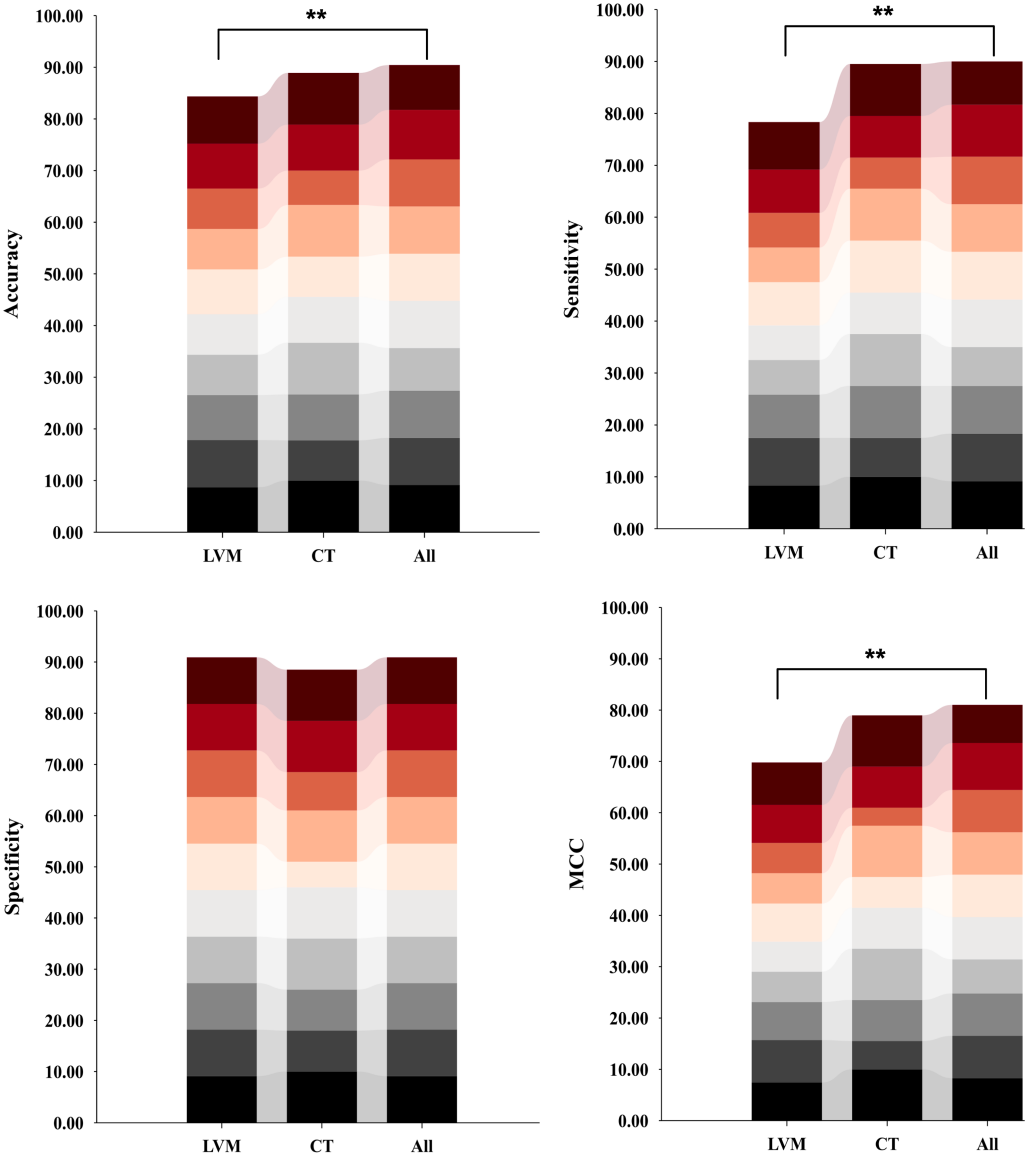
Distribution plots of per-fold classification accuracy, sensitivity, specificity, and MCC results for models constructed based on three different inputs and SVC algorithms on an independent test set. Model performance was compared using ANOVA tests, followed by *post hoc* comparisons (Bonferroni-corrected p < 0.05). Asterisks indicate significant group differences. LVM, lateral ventricular morphometry; CT, cortical thickness measures; All, combined LVM and CT; MCC, Matthews correlation coefficient.

In comparing the model constructed with the All feature integration approach to the one using only the LVM strategy, the former exhibited statistically significant improvement in recognizing iNPH (Figure 5). Specifically, there was an 6.09% increase in accuracy, sensitivity improved by 11.67%, and MCC increased by 11.25%, with a smaller standard deviation.

**Figure 6:**
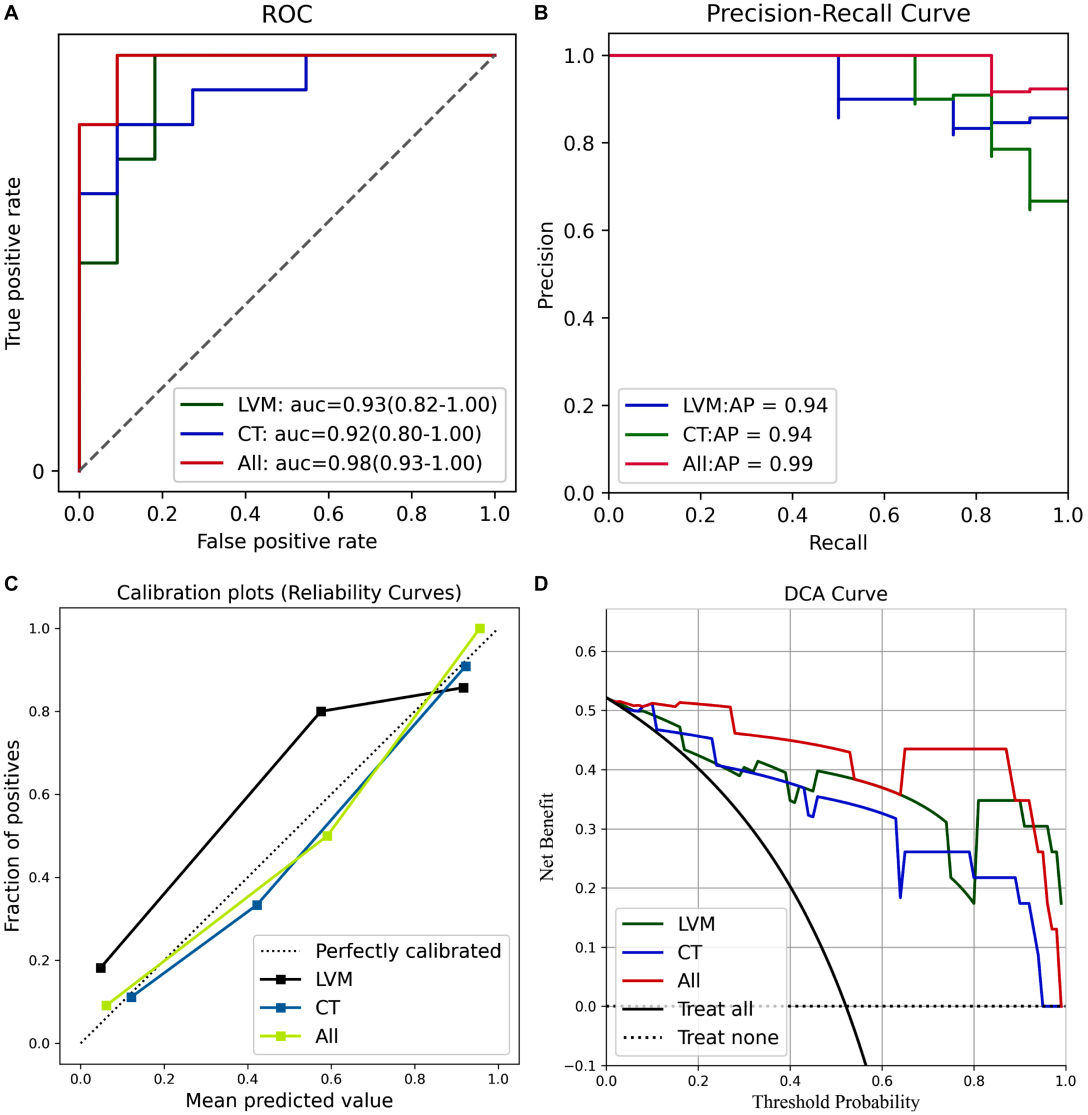
**(A)** ROC curves of the three models. **(B)** PR curves of the three models. **(C)** The calibration curve of the three prediction models. **(D)** The decision curve analysis (DCA) curve of the clinical use assessment of the three prediction models. LVM, lateral ventricular morphometry; CT, cortical thickness measures; All, combined LVM and CT. auc, area under the curve; AP, average precision.

The average predictive performances of the three models in the differential diagnosis of iNPH and HC were demonstrated by plotting the ROC, PR curves, calibration curves, and DCA curves (Figure 6). The area under the curve (AUC) for the All model was 0.98 [95% CI 0.93–1.00], which was higher than the AUC for the LVM model (AUC 0.93 [95% CI 0.82–1.00]) and the CT model (AUC 0.92 [95% CI 0.80–1.00]) (Figure 6A). Although the Delong’s test revealed no significant differences when comparing the AUC of the All model to the LVM model (Z = 0.908, *p*-value = 0.363) and the All model to the CT model (Z = 1.082, *p*-value = 0.279), the combined model (ALL) showed better performance than the LVM or CT models alone. The PR curve also showed that the All model had the best performance, with an Average Precision (AP) value of 0.99 (Figure 6B). Calibration curves were used to demonstrate the agreement between predicted risks and actual observed results. Figure 6C exhibited improved concordance between the forecasted and real probabilities of the All model. Moreover, an intuitive DCA plot was employed to demonstrate the overall advantages of the potential population across various risk thresholds and assess the extent to which the ultimate All model could assist in clinical treatment strategies. When the risk thresholds varied from 0 to 1, the All model achieved the highest net benefit compared with the “treat all” and “treat none” strategies and with the LVM and CT models (Figure 6D).

### 3.5 Feature importance analysis

**Figure 7:**
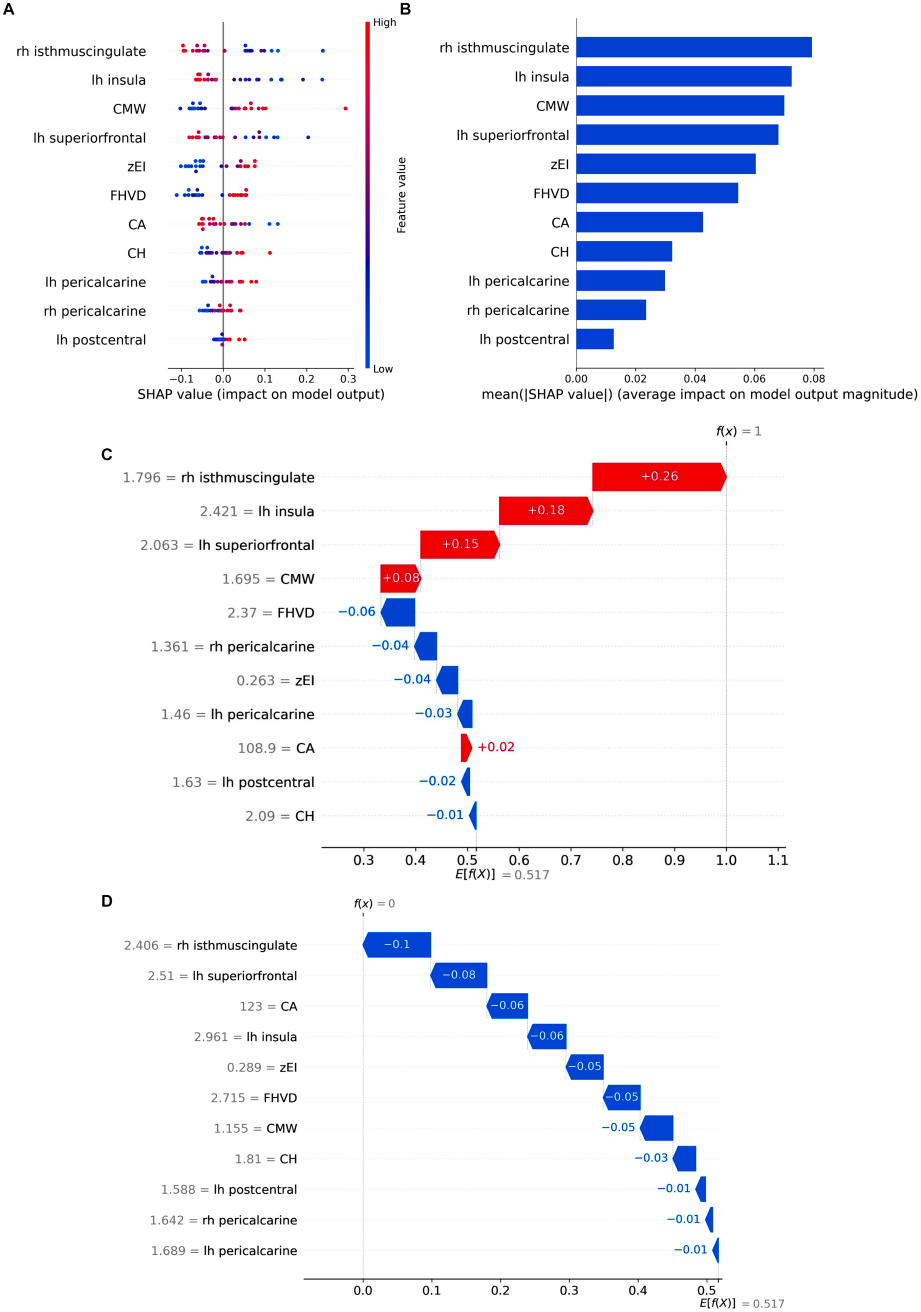
Model Interpretation by SHAP analysis. **(A)** Attributes of features in SHAP. Each line represents a feature, and the abscissa is the SHAP value. Red dots represent higher eigenvalues, and blue dots represent lower eigenvalues. **(B)** Feature importance ranking as indicated by SHAP. The matrix diagram describes the importance of each covariate in the final prediction. The *x*-axis depicts SHAP values that represent the importance of the representative feature. **(C,D)** SHAP waterfall plots of two instance labels predicting. Label “1” represents the iNPH. Label “0” represents the HC. Red lines indicate a positive effect on the predicted outcome. Blue lines indicate a negative effect on the predicted outcome. z-EI, z-evans index, CA, callosal angle; FHVD, frontal horn vertical diameter; CMW, cella media width; CH, callosal height; lh, left hemisphere; rh, right hemisphere.

Shap is utilized to visually demonstrate how the variables contribute to the model’s prediction of iNPH, effectively explaining the role of each feature. In the best All model, Figure 7A displayed 11 key attributes. Different colored dots are used to plot all patient attributions to outcomes on each line of feature importance. Red dots indicate high risk values while blue dots indicate low risk values. The risk of developing iNPH is higher with an increase in CMW, zEI, FHVD and CH, a decrease in CA values, atrophy of the right isthmus cingulate cortex, left insula cortex, and left superior frontal cortex, and thickening of the left pericalcarine region, right pericalcarine area and left postcentral gyrus cortex.

The mean absolute SHAP values were calculated to determine each feature’s importance ranking, represented in Figure 7B. The cortical thickness of the right isthmus cingulate cortex emerged as the most crucial factor in distinguishing iNPH from HC, followed by the cortical thickness of the left insula cortex and CMV value. Notably, morphological metrics such as EI and CVD no longer dominated in the classification process, implying that cortical thickness-based features can potentially replace certain manual measurements and prove more effective for iNPH discrimination. SHAP waterfall plot showed the contribution of individual features when predicting instance labels (Figures 7C,D).

### 3.6 Associations of LVM and CT features with the clinical performance of iNPH

**Figure 8:**
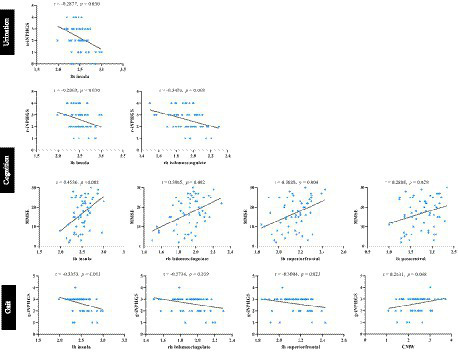
Correlation analysis between LVM and CT features with the clinical performance of iNPH. iNPHGS, idiopathic normal-pressure hydrocephalus grading scale; u-iNPHGS, urinary control-iNPHGS; r-iNPHGS, cognitive-iNPHGS; g-iNPHGS, gait-iNPHGS; MMSE, mini-mental status examination; CMW, cella media width; lh, left hemisphere; rh, right hemisphere.

Correlation analyses were carried out to test whether LVM and CT features were associated with clinical presentation. As shown in Figure 8, the urination performance measured by the u-iNPHGS, showed a marginally significant negative correlation with thinned cortex in the left insula cortex (*r* = −0.287, *p* = 0.030). Both the c-iNPPHGS score and the MMSE score, which assess cognitive function, had significant correlations with cortical thickness of the left insula cortex and right isthmus cingulate cortex (*r*1 = −0.287, *p*1 = 0.030; *r*2 = −0.3478, *p*2 = 0.008; *r*3 = 0.4536, *p*3 < 0.001; *r*4 = 0.396, *p*4 = 0.002;). In addition, there were also a significant positive correlation between MMSE score of iNPH patients and the cortical thickness in the left superior frontal cortex and left postcentral (*r*1 = 0.369, *p*1 = 0.004; *r*2 = −0.289, *p*2 = 0.029). Regarding gait performance, higher cortical thickness in left insula cortex, right isthmus cingulate cortex, and left superior frontal cortex were associated with worse motion function (*r*1 = −0.335, *p*1 = 0.011; *r*2 = −0.273, *p*2 = 0.039; *r*3 = 0.300, *p*3 = 0.023). In the analysis of correlations between LVM morphological properties and clinical present, there was no significant results except for the CMW and the g-iNPHGS score (*r* = 0.263, *p* = 0.048). The above-mentioned results may indicate an more association between the worsening of clinical present and cortical structural changes.

## 4 Discussion

This study delved into the cortical structural changes in patients with iNPH through the use of cortical thickness analysis, aiming to improve the understanding of the pathomechanisms of this condition. Another objective was to assess whether these cortical structural changes contribute to improve the diagnostic performance of iNPH and explore potential association with clinical presentation. The finding that ventricular dilatation can trigger changes in the brain parenchyma provides a new perspective for diagnosis and treatment in iNPH. Utilizing advanced machine learning algorithms to construct classification models, we systematically analyzed and identified features of diagnostic value for iNPH. This is the first study that combined traditional ventricular morphometry with brain parenchymal alterations in iNPH cohorts to construct a model with auxiliary diagnostic value. Our results highlight that (1) cortical structures in specific brain regions are altered in iNPH patients and associated with lateral ventricular expansion; (2) the iNPH diagnostic model constructed based on the fusion feature integration of LVM and CT improves accuracy and sensitivity in the identification of iNPH, with cortical thickness features in specific brain regions proving more effective for identification of iNPH; (3) there is a significant correlation between clinical symptoms and cortical thickness alterations, providing clinicians with additional references in the diagnosis and treatment of iNPH.

Individuals diagnosed with iNPH demonstrated substantial reduction in the bilateral insula, fusiform gyrus, frontal cortical areas, and cingulate cortex, consistent with previous studies (Moore et al., 2012; Kang et al., 2018). Cortical structural changes in the cingulate region had also been reported in the study by Bianco et al. (2022). The precise reason behind its cortical thinning or thickening remains incompletely comprehended. In patients with iNPH, a study found a connection between the thinning of both the orbitofrontal cortex and rostral anterior cingulate gyrus, as well as the enlargement of the ventricular surface. The study also proposed that the expansion of the ventricles might contribute to the involvement of the cortex (Kang et al., 2018). This hypothesis was further examined in the current study. The correlation analysis between morphological indices of the lateral ventricles and cortical thickness values showed that ventricular enlargement was strongly and positively associated with cortical thinning in the insula on both sides, superior frontal cortex on both sides, and left fusiform gyrus. However, it was significantly and negatively correlated with cortical thickening in the pericardium on both sides and the posterior central region on the right side. Among them, cortical atrophy in rh insula, lh superiorfrontal and isthmuscingulate showed the strongest correlation with the morphological indicators of lateral ventricles, such as EI, zEI, FHVD, CMW, CCD, and CH. Cortical regions can be impacted by various mechanisms of ventricular enlargement. According to multiple studies, it has been demonstrated that the expansion of the lateral ventricles exerts pressure on the white matter pathways, particularly the corticospinal tracts and corpus callosum (Siasios et al., 2016; Caligiuri et al., 2022). In cases of axonal damage, neuronal degeneration progresses both distally (known as Wallerian degeneration) and proximally (referred to as apoptosis). Therefore, it is plausible that white matter damage could lead to cortical areas becoming thinner and experiencing a reduction in volume (Kang et al., 2020). Additionally, suggestions have been made that cortical involvement in patients with iNPH may occur as a result of impaired vascular supply caused by distortion or compression of the brain parenchyma, as indicated by studies (Owler and Pickard, 2001; Marshall et al., 2017; Kang et al., 2018). However, a comprehensive understanding of the underlying biological processes remains incomplete. Further research is necessary to thoroughly investigate this aspect and validate these hypotheses.

Additionally, there were thickening in cortical regions at the central high convexity and pericalcarine cortex. The probable reasons behind the thickening of the cerebral high convex cortex in iNPH patients were speculated to be a result of hyperperfusion, early compensation of neural activity, and localized neuroinflammation, leading to reactive gliosis characterized by the activation, proliferation, and growth of glial cells. In Kang’s study, it was observed that patients with iNPH exhibited thickening of the cerebral hyperprojection cortex and corresponding characteristic hyperperfusion pattern (Kang et al., 2020, 2023). Hyperperfusion suggested early compensatory activity enhancement of neuronal population and local neuroinflammation of brain region, while reactive gliosis was a manifestation of inflammation. It is characterized by the activation, proliferation and hypertrophy of glial cells (Gao et al., 2013; Ferraro et al., 2018). This phenomenon has been seen in degenerative conditions where Aβ amyloid deposits in the brain trigger inflammatory responses, resulting in swelling and increased cell numbers (Montal et al., 2018). An increase in cortical volume and cortical thickness associated with glial cell activation and Aβ amyloid deposition can be observed in AD patients in the preclinical stage (Johnson et al., 2014; Femminella et al., 2019; Vilaplana et al., 2020), further verifying the relationship between pathological damage and changes in cortical structure. On the other hand, it has been verified that iNPH patients have damaged glial lymphatic system (Reeves et al., 2020), leading to accumulation of metabolic waste, including amyloid proteins, which may activate microglia and contribute to increased gray matter volume (Maezawa et al., 2011). In addition, corticoreactive astrocyte proliferation and microglial cell proliferation caused by hydrocephalus pulling the brain parenchyma have also been reported (Miller and McAllister, 2007). Notably, our study discovered increased thickness in bilateral parietaloid cortex among iNPH patients, a novel finding that emphasizes the importance of further exploration at the cortical level.

To the best of our knowledge, this is the first study to investigate the diagnostic effectiveness of combined cortical thickness and ventricular dilatation morphologic features for iNPH. First, the simple LR algorithm was employed to build models, and AUC values were calculated on the independent test set to evaluate the diagnostic performance of each single feature for iNPH. Among various lateral ventricular morphometry measurements, the CMW feature exhibited the best diagnostic performance [AUC: 0.91, 95% CI (CI): 0.78–1.00], followed by CH, CA, and zEI. Consistent with the results presented by Ryska et al. (2020), caudal cranial expansion of the ventricular geometry (ZEI, CMW, FHVD) better discriminates iNPH than laterolateral ventricular enlargement (commonly used EI). In contrast, the diagnostic performance of cortical thickness in the right isthmus cingulate cortex exceeded that of lateral ventricular morphometry [AUC: 0.93, 95% CI (CI): 0.82–1.00]. This suggests that, in iNPH, localized regions may be more sensitive to alterations in cortical thickness than changes in lateral ventricle morphology. Then, considering that multiple feature combinations have complementary information to each other, both the LVM and CT features were combined in this work to improve the diagnosis of iNPH and HC. As shown in the results (Table 3; Figures 4–6), models based on fused features perform better than that based on the single type of feature. In particular, the fusion model (All) exhibited a substantial improvement in average accuracy, sensitivity, and MCC values, with an increase in accuracy by 6.09%, sensitivity by 11.67%, and MCC by 11.25% compared to the LVM strategy. Moreover, the combination model demonstrated superior clinical benefits and application value, as evidenced by the improved agreement between the predicted and actual probabilities, indicated by both DCA analysis and calibration curve analysis. This further validates that multi-feature fusion can improve classification performance.

Shap analysis provided a visualization of how each variable predicted iNPH in the model. Taken together, the risk of iNPH increased with higher CMW, zEI, FHVD, and CH, lower CA values, atrophy of the right isthmus cingulate cortex, left insular cortex, and left superior frontal cortex, and thickening of the left frontal cortex, right pericalcarine area and left postcentral gyrus cortex. Notably, cortical thickness in the right isthmus cingulate cortex outperformed lateral ventricular morphometry in the diagnosis of iNPH. This again suggests that cortical thickness may be a more sensitive disease marker that reflects pathophysiologic changes in the disease. This finding would assist clinicians in improving the identification and detection of iNPH.

It is well known that structural decline (i.e., cortical thinning) in specific regions is associated with clinical decline (Pacheco et al., 2015). Similarly, the present study found that worsening of clinical symptoms in the iNPH cohort was strongly associated with cortical structural abnormalities. First, urinary dysfunction was only correlated with cortical atrophy in the left insula. A previous study based on functional neuroimaging similarly reported that patients with overactive bladder disease showed reduced functional activity in cortical sensory processing areas (e.g., insula) (Walter et al., 2021).The possible explanation is that the insula cortex is involved in emotional processing and autonomic nervous system regulation. When the insula cortex is damaged, it may lead to autonomic nervous system imbalance and bladder dysfunction, and cause symptoms such as frequent and urgent urination. Second, both cognitive and motor functions are associated with changes in the left insular cortex, the right isthmus cingulate cortex, and the left superior frontal cortex. The isthmus of the cingulate cortex is the narrow portion of the cingulate cortex that connects the posterior cingulate cortex to the parahippocampal gyrus. Although the function of the cingulate isthmus is unknown, converging evidence from structural imaging suggests a critical role for the cingulate isthmus cortex in cognitive and emotional integration (Mayblyum et al., 2021; Wei et al., 2021). In addition, a large number of previous studies have emphasized the cortical thickness of the left superior frontal cortex as a core area for higher cognitive functions, including attention, working memory, and cognitive control, all of which may affect gait and balance in older adults (van Iersel et al., 2008; Li et al., 2013; Kraljević et al., 2021). A previous study also has reported that the superior frontal gyrus plays a broad role in gait dysfunction in patients with iNPH (Kang et al., 2020). Compared with morphological measures of ventricular dilatation, cortical structural features are able to reveal pathophysiologic changes in the disease, thus providing a more in-depth interpretation of clinical symptoms. This result re-emphasizes the potential of cortical thickness as a more sensitive marker of disease, which may be of higher value as a diagnostic indicator compared with lateral ventricular dilatation.

However, the current research also comes with several limitations. Firstly, although the study included patients diagnosed with iNPH and healthy controls, the sample size was relatively small. Secondly, the study did not perform differential diagnosis with other cognitively impaired neurodegenerative diseases, and it did not investigate whether these cortical alterations could be specific biomarkers for iNPH prevalence. Thirdly, our analysis solely focused on cortical thickness characteristics and ventricular morphology, without including the utilization of lateral ventricular volumes as conducted in the research conducted by Yun et al. (2023) and the study conducted by Vlasak et al. (2022) utilized phase-contrast MRI characteristics. These features have been shown to have some iNPH recognition ability. Therefore, future studies should consider integrating additional features to construct more accurate models.

## 5 Conclusion

This study reveals the significance of considering cortical structural changes in the diagnosis of iNPH. The diagnostic model based on the fusion of LVM and CT demonstrates improved accuracy and sensitivity in identifying iNPH. Particularly noteworthy is the ability of changes in cortical thickness in specific brain areas, such as the right isthmic cingulate cortex, to effectively distinguish iNPH. This suggests that cortical structural characteristics can serve as potential diagnostic markers for iNPH. The findings would assist clinicians in improving the identification and detection of iNPH, enabling timely shunt surgery for candidates, thereby improving patient prognosis and quality of life.

## Data availability statement

The raw data supporting the conclusions of this article will be made available by the authors, without undue reservation.

## Ethics statement

The studies involving humans were approved by Medical Ethics Committee of Huadong Hospital Affiliated to Fudan University. The studies were conducted in accordance with the local legislation and institutional requirements. The ethics committee/institutional review board waived the requirement of written informed consent for participation from the participants or the participants’ legal guardians/next of kin because Written informed consent for participation was not required for this study in accordance with the national legislation and the institutional requirements.

## Author contributions

YY: Methodology, Software, Validation, Visualization, Writing – original draft, Conceptualization, Data curation. MY: Conceptualization, Data curation, Methodology, Validation, Writing – original draft, Investigation. XL: Conceptualization, Data curation, Writing – original draft. SL: Supervision, Writing – review & editing, Funding acquisition. GL: Supervision, Writing – review & editing, Funding acquisition.

## Footnotes

^1^ https://www.fil.ion.ucl.ac.uk/spm/

^2^ http://dbm.neuro.uni-jena.de/cat/
