# Supplementary material for: Improve the diagnosis of idiopathic normal pressure hydrocephalus by combining abnormal cortical thickness and ventricular morphometry
Source: Front Aging Neurosci. 2024 Feb 29;16:1338755. doi: 10.3389/fnagi.2024.1338755 (PMC10937576; doi:10.3389/fnagi.2024.1338755)
Supplement: Supplementary file 1 [file Data_Sheet_1.docx]

**Supplementary Materials**


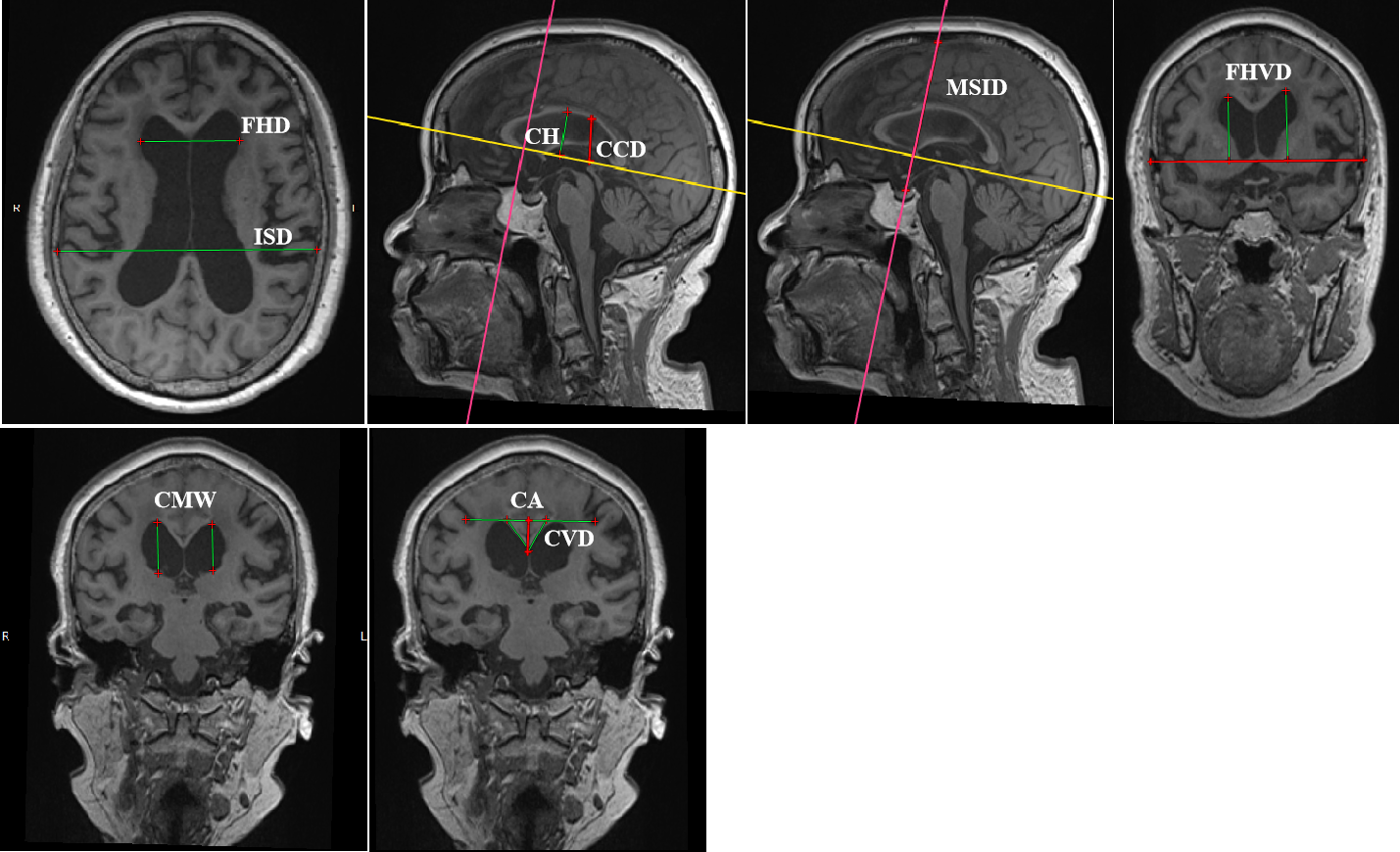


**Figure S1.** The measurement methods of each imaging marker.Frontal hom diameter (FHD) and inner skull diameter (ISD) were measured in the same transverse section. Callosal height (CH) was measured perpendicular to the line of bicallosal line, callosal-commissuraldistance (CCD) parallel to the posterior brain stem margin. Maximumsupratentorialintracranialdiameter (MSID) was measured perpendicular to the bicommissural line. Frontal horn vertical diameter (FHVD) was the distance between the apex of the lateral ventricle. The cella media width (CMW), and temporal horn width (THW) measurements on the coronal section passing through the posterior commissure. Callosal angle (CA) is the angle between the inner wall of the lateral ventricle. Callosal ventricular distance (CVD) was the distance between the line connecting the roofs of the ventricles and the lowest point of the corpus callosum.

**Figure S2.** Feature selection plots for the Lasso regression, which were adjusted by the super parameter (alpha) to determine the optimal features. In the left column (Figure (A), (C), and (E)), the vertical dashed line indicates that the corresponding optimal alpha value when obtaining the minimum deviation value. In the right column (Figure(B), (D), and (F)), the convergence graph displayed the feature coefficients for feature selection by cross-validation. Features with non-zero coefficients were screened out corresponding to the vertical lines in the plot. Figure (A) and (B) pretend to the LVM-based model, which the optimal alpha value was 0.0124, resulting in the selection of six best features. Figure (C) and (D) corresponded to the CT-based model, with an optimal alpha value of 0.0115 and a total of seven best features selected. Figure (E) and (F) represented the All-based model, with an optimal alpha value of 0.0095 and a total of eleven best features selected. LVM, lateral ventricular morphometry; CT, cortical thickness; All, the combination of lateral ventricular morphometry and cortical thickness.


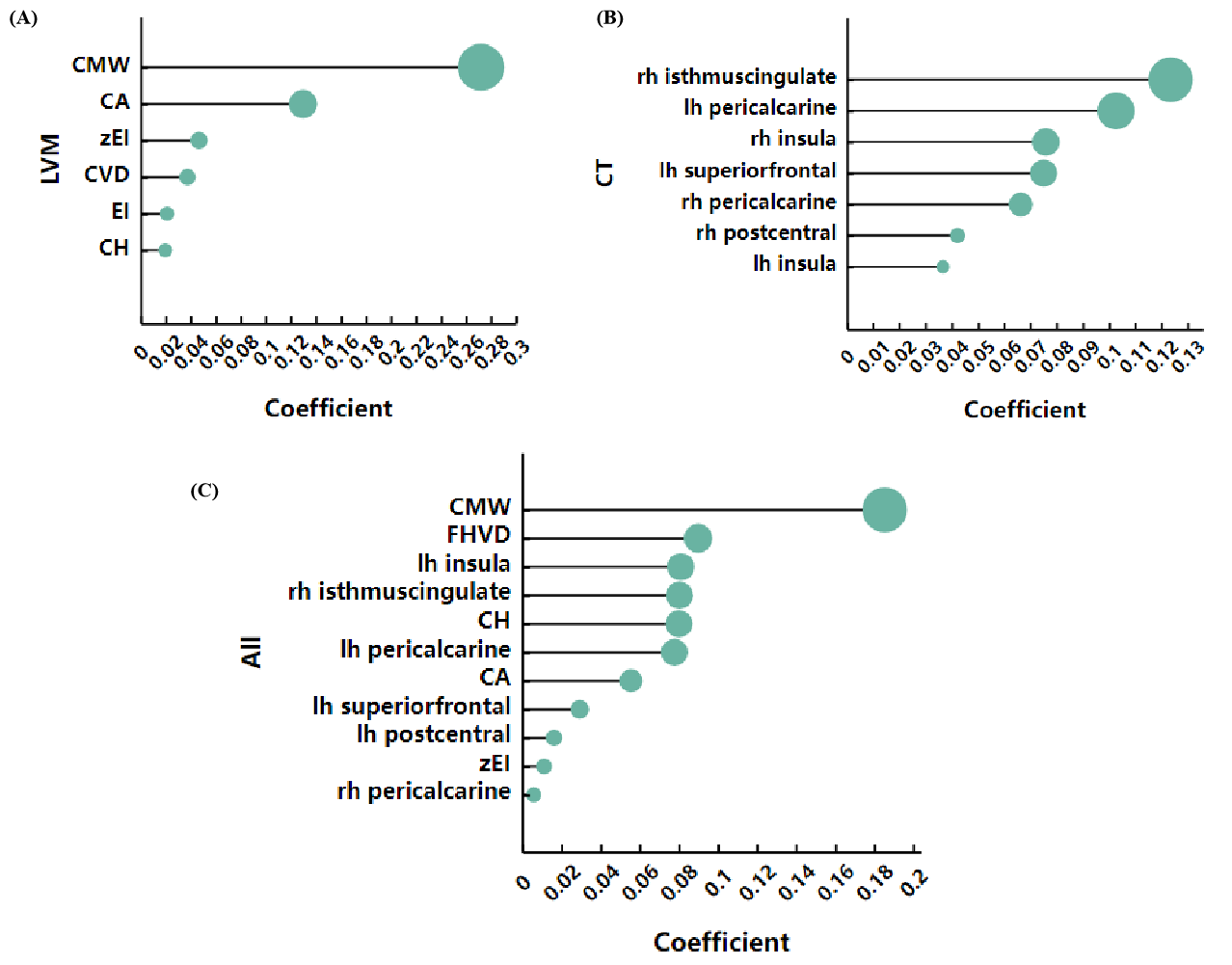


**Figure S3.** Features and weights of LASSO regression screening. LVM, lateral ventricular morphometry; CT, cortical thickness; All, the combination of lateral ventricular morphometry and cortical thickness
